# Video Instance Segmentation Through Hierarchical Offset Compensation and Temporal Memory Update for UAV Aerial Images

**DOI:** 10.3390/s25144274

**Published:** 2025-07-09

**Authors:** Ying Huang, Yinhui Zhang, Zifen He, Yunnan Deng

**Affiliations:** Faculty of Mechanical and Electrical Engineering, Kunming University of Science and Technology, Kunming 650500, China; 20213103003@stu.kust.edu.cn (Y.H.); 20050034@kust.edu.cn (Z.H.); 20203103002@stu.kust.edu.cn (Y.D.)

**Keywords:** video instance segmentation, intelligent inspection, unmanned aerial vehicle (UAV) aerial images

## Abstract

Despite the pivotal role of unmanned aerial vehicles (UAVs) in intelligent inspection tasks, existing video instance segmentation methods struggle with irregular deforming targets, leading to inconsistent segmentation results due to ineffective feature offset capture and temporal correlation modeling. To address this issue, we propose a hierarchical offset compensation and temporal memory update method for video instance segmentation (HT-VIS) with a high generalization ability. Firstly, a hierarchical offset compensation (HOC) module in the form of a sequential and parallel connection is designed to perform deformable offset for the same flexible target across frames, which benefits from compensating for spatial motion features at the time sequence. Next, the temporal memory update (TMU) module is developed by employing convolutional long-short-term memory (ConvLSTM) between the current and adjacent frames to establish the temporal dynamic context correlation and update the current frame feature effectively. Finally, extensive experimental results demonstrate the superiority of the proposed HDNet method when applied to the public YouTubeVIS-2019 dataset and a self-built UAV-Seg segmentation dataset. On four typical datasets (i.e., Zoo, Street, Vehicle, and Sport) extracted from YoutubeVIS-2019 according to category characteristics, the proposed HT-VIS outperforms the state-of-the-art CNN-based VIS methods CrossVIS by 3.9%, 2.0%, 0.3%, and 3.8% in average segmentation accuracy, respectively. On the self-built UAV-VIS dataset, our HT-VIS with PHOC surpasses the baseline SipMask by 2.1% and achieves the highest average segmentation accuracy of 37.4% in the CNN-based methods, demonstrating the effectiveness and robustness of our proposed framework.

## 1. Introduction

In recent years, with their high flexibility, quick response, and cost effectiveness, unmanned aerial vehicles (UAVs) have been widely applied to diverse smart aerial operation domains to identify and analyze emergent challenges [1]. In the civil domain, UAVs are used for wildlife protection [2], traffic monitoring [3,4,5], and forest fire detection [6]. In the military field, UAVs are applied to reconnoiter and monitor battlefields by identifying targets of a small size [6] to achieve a precision attack. Most of the existing methods focus on traditional image-level object detection or instance segmentation to achieve environment awareness through classification, localization, and segmentation for targets. However, the visual systems of UAVs are subject to camera moving speed, shooting angle, and distance from the target location, resulting in the dynamic multiple objects captured suffering from topological deformation, motion blur, scale variation, and heavy occlusion. Traditional image instance segmentation methods segment objects on static individual frames and ignore the temporal correlation between consecutive frames in the video sequence, failing to track the consistent identities of targets. Extending segmentation objects from static, independent images to dynamic, continuous video sequences, the video instance segmentation (VIS) task could simultaneously achieve detection, segmentation, and tracking for instances, which is essential for an intelligent vision system of a UAV to track the consistent identities in the air patrol inspection task. Compared to previous video object segmentation approaches [7,8,9,10,11,12,13,14] focusing on the segmentation of foreground objects from the background, the VIS task aims to establish spatio-temporal correlations for target instances, ensuring consistent target ID tracking across video sequences. This is fundamental for developing intelligent vision systems for UAVs.

Contemporal approaches to video instance segmentation (VIS) can be broadly divided into CNN-based and transformer-based paradigms. Within CNN-based methodologies, the instance tracking framework [15,16,17,18,19] typically employs per-frame instance segmentation followed by temporal post-processing to enhance consistency across frames. While this paradigm demonstrates effectiveness in maintaining instance identity through global context aggregation, its reliance on frame-independent feature extraction renders it susceptible to performance degradation when handling significant appearance variations, scale changes, or complex inter-frame spatial dependencies. In contrast, feature propagation methods [20,21,22,23] have emerged as a promising alternative by exploiting temporal coherence between adjacent frames. These approaches utilize features from preceding frames to guide current frame segmentation, thereby capitalizing on the inherent redundancy in video sequences. For instance, ref. [21] employs optical flow to warp features from keyframes to non-key frames, reducing computational overhead while introducing dependence on the accuracy of keyframe segmentation. To better build inter-frame correlations, ref. [24] introduced an efficient matching mechanism named instance flow to encode a messy temporal correspondence at the instance level. Nevertheless, the explicit optical flow estimation method faces challenges in handling irregular deformations and occlusions, which makes it difficult to obtain accurate motion compensation.

Among transformer-based VIS methods, the frame-level video instance segmentation paradigm [25,26] regards a video sequence as a series of consecutive frames, and then encodes each frame feature independently and correlates cross-frame instance masks through post-processing. IDOL [25] enhances segmentation accuracy by establishing cross-frame associations of the same instance features. However, instance association based on heuristic matching often leads to temporal inconsistency and constrains the performance improvement. To address this, TCOVIS [26] introduces object association through query propagation and incorporates global instance assignment to reinforce temporal coherence. However, the Frame-level VIS method is inevitably limited to capturing global spatio-temporal context information when learning frame-level feature embeddings and relies on post-processed instance correlations, resulting in error propagation. Clip-level video instance segmentation methods [27,28,29,30] encode independent video clips as 3D spatio-temporal embeddings and directly predict per-frame masks for each instance, eliminating the limitations of the frame-level VIS methods that require correlated branching across frames of instances. VisTR [27], SeqFormer [28], and Mask2Former [29] leverage the DETR architecture to establish transformer-based cross-attention for processing 3D spatio-temporal embeddings. By interacting with long and short-term memories to construct contrast terms to facilitate the discriminative power of instance embedding, with noise introduced during memory bank updates, CTVIS [30] achieves excellent segmentation performance. However, clip-level methods necessitate processing entire videos during training or inference, inevitably incurring substantial computational overhead and memory consumption. Despite their superior temporal information association capabilities, transformer-based VIS methods are constrained by computational demands and robustness limitations in unmanned inspection applications. Therefore, the more lightweight CNN-based VIS framework named SipMask is used as the baseline in this study.

However, a great challenge for the video instance segmentation task is that of multiple instances with similar appearances of the same category in the video sequence, which exhibit various magnitudes of geometric deformation at different scales, generally leading to incorrect IDs during the tracking and identification of multiple individuals across frames. Although deformable convolution-based motion compensation methods are utilized to compensate for limited information of objects with complex appearance features and topological variations at frame levels, the narrow receptive field (RF) and a single-level feature extraction approach make it intrinsically less effective against the variety of object appearances and sizes when computing the correlation motion offsets. Motivated by the above observation, we present a hierarchical video instance segmentation framework for UAV aerial images, named HT-VIS. Our main contributions are as follows:

(1) We propose a hierarchical offset compensation (HOC) module in the form of sequential and parallel connection, so as to further obtain adequate geometrical information from surrounding reliable pixels, generating cross-scale deformable offset signals for the flexible multi-scale targets across video frames and compensating for spatial motion features.

(2) A temporal memory update (TMU) module is proposed that employs the ConvLSTM to establish temporal dynamic context relations and update the current frame information effectively.

(3) We divide the public dataset YouTube-VIS 2019 into four different typical UAV scenarios, including Zoo, Street, Vehicle, and Sport, to simulate popular aerial intelligent scenarios. Moreover, we build a dataset from a UAV perspective named UAV-VIS for intelligent traffic scenarios, which is used to further comprehensively validate the performance of the proposed HT-VIS.

(4) On four constructed typical UAV datasets and the self-built UAV-VIS dataset, extensive experiments are conducted to evaluate the effectiveness of the proposed HT-VIS model and demonstrate the improved segmentation quality compared to the state-of-the-art methods.

The remaining parts of this paper are arranged as follows: Section 2 describes related works. Section 3 presents the overall framework with detailed discussions of the HOC and TMU modules. Section 4 provides extensive empirical evaluations and demonstrates the generalization and robustness of the proposed method. Section 5 gives the conclusion. Section 6 presents a discussion and Section 7 provides the limitations and future work.

## 2. Related Work

### 2.1. Video Instance Segmentation

In this paper, we categorize previous works on video instance segmentation into lightweight CNN-based or heavyweight transformer-based frameworks. Among them, the lightweight CNN-based VIS methods either follow instance tracking or mask propagation paradigms. The first paradigm generally correlates the inter-frame segmentation results via the temporal consistency of inter-frame instances, thus eliminating the ambiguity caused by single-frame information. Another one is to exploit the redundancy of frames by propagating the spatial features of an independent frame in a video clip to other frames in the time sequence and then completing the pixel classification by feature interaction.

#### 2.1.1. CNN-Based VIS Methods

Instance Tracking. Ref. [15] transformed the instance tracking task into a multi-classification for associating instances across single frames by tailoring and adding a tracking branch on [31], and first performed a combined task of detecting, tracking, and segmenting instances in video sequences. However, ref. [15] only focused on the feature extraction of a singe frame without utilizing information from other video frames, hence neglecting the critical temporal correlation. SipMask [16] improves upon MaskTrack R-CNN [15] by introducing a compact tracking branch with two convolutional layers to encode instance representations at bounding box centers, but its simple matching matrix struggles with occlusions due to limited temporal context integration. To improve the tracking capability, ref. [18] presented a comprehensive feature aggregation method by designing a new attention mechanism to refine temporal and spatial context features, yet the added attention layers increased extra computational complexity. To improve the tracking capability, ref. [19] introduced a temporal fusion module to infer the displacement of instances between consecutive frames, thereby obtaining spatio-temporal instance masks. Ref. [17] performed segmentation in different sub-regions dynamically divided by instances and achieved richer mask granularity. Nonetheless, the reference frame is randomly selected based on the current frame, which tends to confuse the time domain information during the training process. The instance tracking-based approaches are not only independent of the video sequence length but also reuse the feature extraction paradigm of existing image instance segmentation methods to encode features frame-by-frame, followed by cross-frame correlation. However, the matching strategy of heuristic association is difficult to learn for dynamically variable deformation instances, and the frame-level feature processing fails to capture the consistency of target motion.

Mask Propagation. By developing a mask propagation branch on Mask R-CNN, ref. [20] propagated frame-level instance masks from the current frame to the remaining frames in video clips. Although the segmentation accuracy was improved, the offline learning method of MaskProp led to a large memory occupation and a long training period. Ref. [21] defined key frames and reference frames according to the redundancy of video clips, where the high-level features of the reference frame are propagated by the previous key frame to reduce unnecessary spatial–temporal complexity and improve the segmentation speed. However, this propagation relies on the accuracy of the deep features of the defined keyframes, and poor keyframe segmentation results in an accumulation of errors in the segmentation task for non-keyframes. Ref. [23] directly modeled video clips as a 3D spatio-temporal embedding to cluster pixels over the entire video sequence, resulting in high computational complexity. Ref. [22] builds a crossover learning strategy upon the instance segmentation network [32], so as to pixel-wisely localize the same instance in other frames, which introduces cost-free enhancement during inference. Mask propagation-based approaches significantly improve timing consistency by explicitly propagating the masks or features of reference frames. However, this paradigm is limited by the mask quality of the key frames and introduces an increased inference time when dealing with long-time video sequences.

#### 2.1.2. Heavyweight Transformer-Based VIS Methods

In the domain of video instance segmentation, offline approaches aim to associate spatio-temporal features across video clips for a high-quality mask of instances. Online VIS methodologies typically follow a “segment by associate” paradigm.

Offline methods. VITA [33] efficiently establishes instance relationships by correlating frame-level object tokens, enabling holistic video-level understanding. SeqFormer [28] introduces a Query separation mechanism through generating distinct features for each object in an individual frame and then aggregating these features in the global dimension, so as to represent each instance more efficiently at the video level. The offline method jointly exploits the instance masks of all frames to avoid frame-by-frame error accumulation and significantly improves cross-frame consistency. However, it is necessary to process the complete video clip at once, and capturing long-range dependencies using Transformer or 3D convolution leads to excessive computational complexity.

Online methods. IDOL [25] innovatively presents an online framework based on contrastive learning, which enhances segmentation stability and consistency by learning discriminative instance embeddings and exploiting the features of historical frames to establish cross-frame associations. While effective in maintaining temporal coherence, its reliance on sequential processing increases latency in long videos. MinVIS [34] applies a query-based image instance segmentation method to each video frame and then tracks instances through binary matching. Although MinVIS reduces the computational complexity and improves the segmentation accuracy, it still has limited robustness in long video instance matching. To learn highly discriminative instance embedding features, CTVIS [30] introduces a momentum-averaged embedding and memory bank storage mechanism to achieve a reliable comparison between the current instance embeddings and the stable representation of historical instances, thereby enhancing the quality of mask segmentation of instances. However, improving the quality of the mask by matching current instances with the memory bank leads to additional storage space. In conclusion, although the transformer method improves the segmentation accuracy of the model, the computational redundancy of the multi-head self-attention mechanism leads to increased computational complexity, which is not applicable in UAV inspection scenarios with lightweight deployment.

### 2.2. Deformable Convolution for Video Tasks

Deformable convolution networks (DCNs) [35] were initially proposed to model geometrically transformed objects in images through dynamic filtering and have since been widely adopted in various computer vision tasks, including video super-resolution [36,37], video object detection [38], and pose estimation [39]. Specifically, ref. [36] introduced feature-level alignment of consecutive frames using deformable convolution, eliminating the need for explicit optical flow estimation. Building upon this, ref. [37] proposed a cascaded deformable alignment module following a pyramid structure to progressively refine the features of input frames. For video object detection, ref. [38] employed a spatio-temporal sampling mechanism based on deformable convolution to propagate object features across frames. Similarly, ref. [39] leveraged deformable convolution to implicitly warp features from sparsely labeled frames to neighboring unlabeled frames in a temporal sequence. Ref. [20] introduced an implicit feature propagation mechanism by predicting per-pixel offsets between adjacent frames, achieving robust performance under challenging conditions such as occlusion and large motion.

## 3. Method Overview of HT-VIS

Given a video sequence $I\;=\;{\left.\left\{I_{t}\right.\right\}}_{t=1}^{T}$ with *T* input frames, we define the previous frame $I_{t}\in R^{3\times H\times W}$ adjacent to the current $I_{t}$ as the reference frame, where *H* and *W* represent the initial size of the input frame. The goal of HT-VIS is to estimate and compensate hierarchically the spatial motion offsets in time series for moving targets and densely track instances with consistent identities.

**Figure 1:**
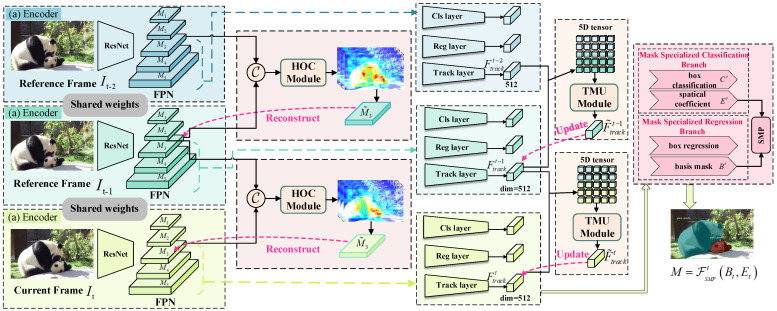
HT-VIS network architecture. (Pink arrows indicate the reconstructed features. Blue, green and yellow arrows indicate the FPN features of the reference frame $I_{t-2}$, the reference frame $I_{t-1}$ and the current frame $I_{t}$, respectively).

The overall pipeline in our approach is illustrated in Figure 1 and mainly involves three key components: (a) Encoder, (b) HOC, and (c) TMU. Specifically, the current and reference frames are first fed into a shared backbone encoder, which employs ResNet50, consisting of four residual blocks, to extract frame features, followed by the feature pyramid network (FPN) for richer representations $M={\left\{M_{l}\in R^{256\times H_{l}\times W_{l}}\right\}}_{l=1}^{5}$ at *l*-th the layer. After that, the cross-channel concatenated layers $C\left(M_{l}^{t-1},M_{l}^{t}\right)$ are passed through our proposed HOC module to generate spatio-temporal features to compensate for the missing details and dynamically reconstruct the ${\tilde{M}}_{l}^{t}$ layer, where $C\left(\cdot ,\cdot \right)$ indicates cross-channel concatenation. Then, the resulting features of the current and reference frame at the *l*-th level (l=3,4,5) are integrated to separately obtain the tracking features $F_{track}^{t}=F_{conv}\left(C\left({\tilde{M}}_{3}^{t},M_{4}^{t},M_{5}^{t}\right)\right)\in R^{256\times H_{5}\times W_{5}}$ and $F_{track}^{t-1}=F_{conv}\left(C\left({\tilde{M}}_{3}^{t-1},M_{4}^{t-1},M_{5}^{t-1}\right)\right)\in R^{256\times H_{5}\times W_{5}}$ by using track layers consisting of two convolution layers. Additionally, such tracking feature maps of $I_{t}$ and $I_{t-1}$ are applied to the TMU module to establish the temporal context relationship and update the current frame feature $F_{track}^{t}=F_{TMU}\left(F_{track}^{t},F_{track}^{t-1}\right)$ effectively. Meanwhile, the powerful fused features at the *l*-th level of (l=3,4,5) are simultaneously transmitted into the prediction heads composed of a mask-specialized classification branch and a mask-specialized regression branch, which are in parallel to the tracking branch. The mask-specialized classification branch aims to generate class confidence scores $C^{t}\in \left[0,1\right]$ and spatial coefficients $E^{t}\in R^{m\times e\times e\times p}$ for e×e sub-regions in predicted boxes of *p*, where *m* denotes the predicted basis mask. Then, the corresponding instance mask $B^{t}\in R^{H\times W\times m}$ can be obtained by the mask-specialized regression branch for the whole image. Finally, the mask predictions $M=F_{SMP}^{t}\left(B_{t},E_{t}\right)$ for the current frame are output by the spatial mask prediction (SMP) module.

### 3.1. Hierarchical Offset Compensation Module

Previous work [36,37,38,39] has demonstrated that deformable convolution is capable of performing video-level alignment and frame interpolation. Nonetheless, these approaches using limited RF or singe-level feature extraction face challenges in capturing hierarchical motion offsets for multi-scale moving targets that are continuously undergoing topological deformation, especially for the same class. In this work, a HOC module is proposed aided by deformable convolution. Compared to previous motion offsets estimation strategies, HOC has two advantages: (1) $n\in \left\{1,2,3,4\right\}$ spatial motion offset (SMO) mechanisms corresponding to different dilation factors $r\in \left\{3,6,9,12\right\}$ are developed to enlarge the RF range of used motion information for multi-scale instances across frames. When *r* increases, each local grid feature can be well captured by the central pixel to obtain the motion feature of the large-scale target. Meanwhile, small *r* facilitates the accurate capture of small target motion by focusing on a small region. (2) Depending on the paradigm of the SMO mechanisms, the hierarchical offset compensation is further divided into sequential (SHOC) and parallel (PHOC). In the SHOC module, the previous motion feature results with limited RF are as the guidance for the next SMO with richer ones, to incrementally acquire a more substantial set of motion features. In the PHOC module, the multi-scale aggregation strategy is exploited to building dense correlation maps, which ensures more accurate offset estimation.

At the l−th level of the feature pyramid network, the features of the reference frame $I_{t-1}$ and the current frame $I_{t}$ are fused to obtain a temporal feature $F_{l,in}^{t-1\to t}$ and are used as input to the HOC module. This fused temporal feature $F_{l,in}^{t-1\to t}$ contains the feature mapping from moment t−1 to moment *t*. The HOC module achieves spatial motion compensation by calculating the motion offset between adjacent frames through deformable convolution. Given a deformable convolution $D_{r}^{p}\left(\cdot \right)$ with padding *p* and dilation rate *r*, the calculation formula can be expressed as Equation (1).

(1) $$F_{l,out}^{t-1\to t}\left(f_{l,0}\right)=\sum_{f_{l,r}\in \Theta_{r}}\omega \left(f_{l,r}\right)\cdot F_{l,in}^{t-1\to t}\left(f_{l,0}+f_{l,r}+\Delta f_{l,r}^{t-1\to t}\right)$$

where $f_{l,0}$ denotes the centre of the sampling location with an offset of 0 and $f_{l,r}\in \Theta_{r}$ denotes the sampling position with an $r^{2}$ in a r×r regular grid of standard convolutions. $\Delta f_{l,r}^{t-1\to t}$ denotes a learnable offset that shifts the sampling points off the standard grid, enabling the adaptation of the target’s irregular shape from time step t−1 to *t*. ω(·) indicates the weight assigned to each sample point of the convolution kernel. $F_{l,out}^{t-1\to t}$ denotes the output feature after deformable convolution corresponding to the position of the sampling centre $f_{l,0}$. $\Theta_{r}$ is a point of the 3×3 grid in our proposed method represented by Equation (2). The symbolic variables in Section 3.1 are defined as shown in Table 1.

(2) $$\Theta_{r}=\left\{\left(-r,-r\right),\left(-r,0\right),\left(-r,r\right),\ldots ,\left(r,0\right),\left(r,r\right)\right\}$$

The receptive fields for per sampling points become richer as the dilation rate is from 3 to 12, which adjusts the shape of the convolution kernel, and capture multi-scale deformation features of the targets. In addition, four spatial motion offset mechanisms are designed to explore the offset $\Delta f_{l,n}^{t-1\to t}$ between two input frames with different dilation rates, each of which consists of a standard convolution filter $\Lambda_{r}^{p}\left(\cdot \right)$ and a deformable convolution $D_{r}^{p}\left(\cdot \right)$ with k×k×256, where padding *p* is set to the same value as dilation rate *r* in each SMO module $n\in \left\{1,2,3,4\right\}$; $D_{r}^{p}\left(\cdot \right)$ denotes the deformable convolution described in Equation (1).

**Figure 2:**
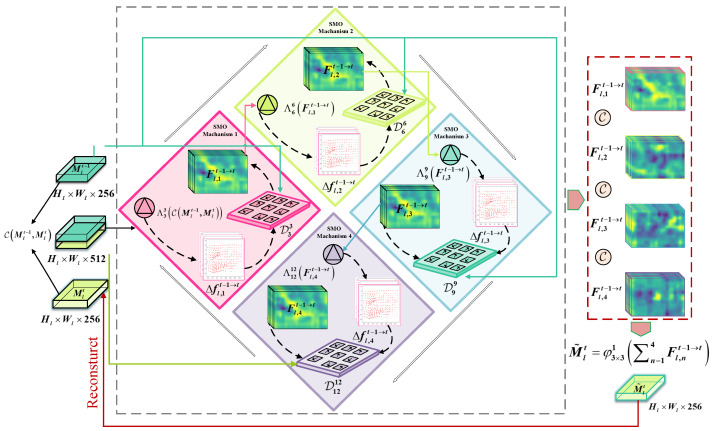
Structure of SHOC module. (Black arrows represent the feature flow from SMO mechanism 1 to SMO mechanism 2. Cyan arrows indicate the feature of reference frame, yellow arrows indicate the feature of current frame).

The SHOC module is shown in Figure 2; the previous compensation features of SMO are regarded as a guidance to carry out more abundant offsets. In detail, a convolution filter with $k\times k\times 2k^{2}$ is first utilized to predict motion offsets $\Delta f_{l,1}^{t-1\to t}=\Lambda_{r}^{p}\left(C\left(M_{l}^{t-1},M_{l}^{t}\right)\right)\in R^{H_{l}\times W_{l}\times 18},r=3$ for the concatenated features $C\left(M_{l}^{t-1},M_{l}^{t}\right)\in R^{H_{l}\times W_{l}\times 512}$. Then, the deformable convolution with p=3 takes such offsets and the $M_{l}^{t-1}$ layer of the reference frame as input to produce the compensation features of the current frame, denoted by:

(3) $$F_{l,1}^{t-1\to t}=D_{r}^{p}\left(\Delta f_{l,1}^{t-1\to t},M_{l}^{t-1}\right)\in R^{H_{l}\times W_{l}\times 256},r=3$$

Differing from the first SMO mechanism, when $n\in \left\{2,3\right\}$, we generate the feature $F_{l,n}^{t-1\to t}=D_{r}^{p}\left(\Delta f_{l,n}^{t-1\to t},M_{l}^{t-1}\right)\in R^{H_{l}\times W_{l}\times 256},r\in \left\{6,9\right\}$ by the offset $\Delta f_{l,n}^{t-1\to t}=\Lambda_{r}^{p}\left(F_{l,n-1}^{t-1\to t}\right)\in R^{H_{l}\times W_{l}\times 18}$ and the $M_{l}^{t-1}$ layer of the reference frame, where $F_{l,n-1}^{t-1\to t}$ is the output feature of the previous $\left(n-1\right)-th$ SMO. At the last module, the compensation feature $F_{l,4}^{t-1\to t}$ is obtained by $F_{l,3}^{t-1\to t}$ and the $M_{l}^{t}$ layer at step *t*:

(4) $$F_{l,4}^{t-1\to t}=D_{r}^{p}\left(\Lambda_{r}^{p}\left(F_{l,3}^{t-1\to t}\right),M_{l}^{t}\right),r=12$$

Finally, the multi-scale features of four SMO mechanisms are hierarchical concatenated along the channel dimension, followed by a standard convolution layer $\varphi_{3\times 3}^{s}\left(\cdot \right)$, where *s* refers to stride for the 3×3×256 filter to refine the output feature map ${\tilde{M}}_{l}^{t}\in R^{H_{l}\times W_{l}\times 256}$ and reconstruct the $M_{l}^{t}$ layer of the current frame:

(5) $${\tilde{M}}_{l}^{t}=\varphi_{3\times 3}^{1}\left(\sum_{n=1}^{4}F_{l,n}^{t-1\to t}\right)$$

**Figure 3:**
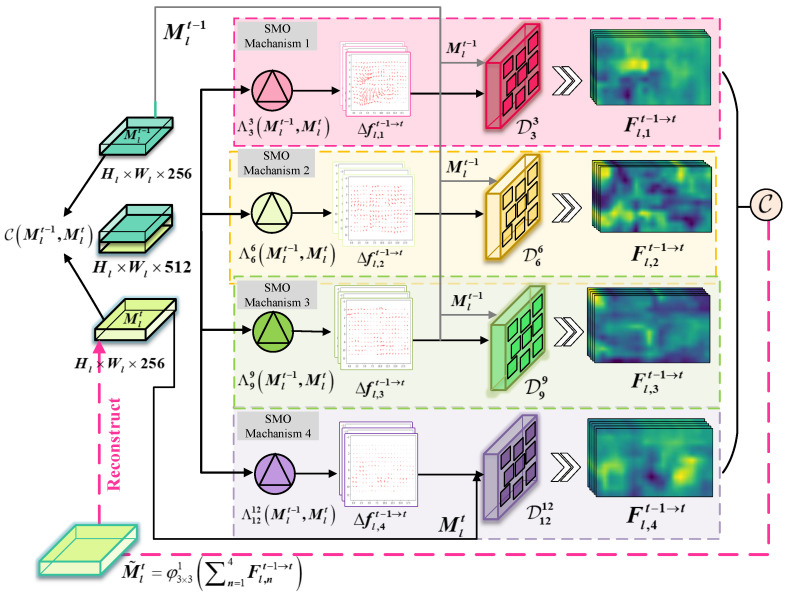
Structure of PHOC module. (The pink, yellow, greeen, and purple areas represent the SMO mechanism 1, 2, 3, and 4, respectively. Black arrows indicate the directions of feature flow, pink dashed arrows denote the reconstructed feature output by PHOC module).

As illustrated in Figure 3, four SMO mechanisms are applied in the PHOC module to produce a set of hierarchical offset features with the same dimensions in parallel: $\Delta f_{l,n}^{t-1\to t}=\Lambda_{r}^{p}\left(C\left(M_{l}^{t-1},M_{l}^{t}\right)\right)\in R^{H_{l}\times W_{l}\times k^{2}},r\in \left\{3,6,9\right\}$. Then, the offset features of ${\left\{\Delta f_{l,n}^{t-1\to t}\right\}}_{n=1}^{3}$ are fed into corresponding deformable convolution filters to learn the importance of different scales simultaneously and produce three parallel spatio-temporal features: $F_{l,n}^{t-1\to t}=D_{r}^{p}\left(\Delta f_{l,n}^{t-1\to t},M_{l}^{t-1}\right),r\in \left\{3,6,9\right\}$. At the last module, the spatial motion offset is generated by:

(6) $$F_{l,4}^{t-1\to t}=D_{r}^{p}\left(\Delta f_{l,4}^{t-1\to t},M_{l}^{t}\right),r=12$$

Finally, a similar fusion operation is conducted following SHOC to integrate the hierarchical motion offset feature as Equation (4).

### 3.2. Temporal Memory Update with ConvLSTM

As video is a sequence of frames with large temporal content variance, the performance of the video instance segmentation task is generally subject to object occlusion and scale variation in highly diverse and unstructured videos. To address the issue of tracking target loss or segmentation errors, it is natural to establish correlations between individual frames in instances across video sequences with a constant posture. Therefore, a module termed temporal memory update is designed in this paper by using ConvLSTM to model the dynamic context temporal dependency between the current frame and historical adjacent sequences in a video.

**Figure 4:**
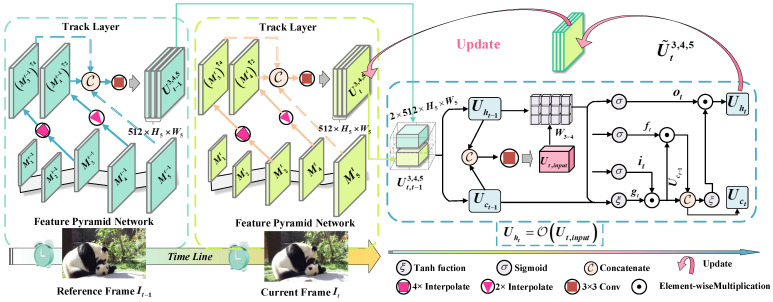
Process of TMU module.

Figure 4 describes the proposed temporal memory update module; the correlation feature from the reference frame $I_{t-1}$ is transmitted to accurately predict the segmentation of the current frame. Specifically, we first concatenate features at the ${\left\{M_{l}\right\}}_{l=3}^{5}$ layers to obtain the multi-modal feature map $U_{t}^{3,4,5}\in R^{768\times H_{5}\times W_{5}}$ at the time step t and generate the $U_{t-1}^{3,4,5}\in R^{768\times H_{5}\times W_{5}}$ for previous frame $I_{t-1}$; then, a 1×1 convolution $\varphi_{1\times 1}^{1}\left(\cdot \right)$ with stride 1 is utilized to integrate non-linear interaction for $U_{t}^{3,4,5}\in R^{512\times H_{5}\times W_{5}}$ and $U_{t-1}^{3,4,5}\in R^{512\times H_{5}\times W_{5}}$ across channel features, respectively:

(7) $$U_{t}^{3,4,5}=\varphi_{1\times 1}^{1}\left(C\left({\left(M_{3}^{t}\right)}^{↑4},{\left(M_{4}^{t}\right)}^{↑2},M_{5}^{t}\right)\right)$$

where ${\left(\cdot \right)}^{↑z}$ represents upscaling with factor *z*. After that, the generated results are stacked up in chronological order as a temporal feature $U_{t,t-1}^{3,4,5}\in R^{2\times 512\times H_{5}\times W_{5}}$ and split into a hidden layer $U_{h_{t-1}}\in R^{512\times H_{5}\times W_{5}}$ as well as cell state feature $U_{c_{t-1}}\in R^{512\times H_{5}\times W_{5}}$ at time step *t*. Then, a 3×3 convolution layer $\varphi_{3\times 3}^{1}\left(\cdot \right)$ is applied to produce input features for the ConvLSTM $O\left(\cdot \right)$ unit:

(8) $${U_{t,}}_{input}=\varphi_{3\times 3}^{1}\left(C\left(U_{h_{t-1}},U_{c_{t-1}}\right)\right)$$

The ConvLSTM unit includes four key components: an input gate $i_{t}$, an forget gate $f_{t}$, a candidate cell state $C_{t}$, and an output gate $O_{t}$. At each time step *t*, the interaction among cell state and three gates is updated as:

(9) $${\left[\begin{array}{cccc} f_{t} & i_{t} & o_{t} & g_{t} \end{array}\right]}^{T}={\left[\begin{array}{cccc} \sigma & \sigma & \sigma & \xi \end{array}\right]}^{T}W_{4\times 3}{\left[\begin{array}{ccc} {U_{t,}}_{input} & U_{h_{t-1}} & 1 \end{array}\right]}^{T}$$

where $\sigma \left(\cdot \right)$ is a sigmoid transfer function and $\xi \left(\cdot \right)$ is hyperbolic tangent non-linear activation function, $W_{3\times 4}$ represent a weights matrix of 3×4 dimension for three gates. Afterwards, the output hidden state $U_{h_{t}}=o_{t}⊙\tanh\left(U_{c_{t}}\right)$ obtained by the updated cell state $U_{c_{t}}=f_{t}⊙U_{c_{t-1}}+i_{t}⊙g_{t-1}$, where ⊙ is an element-wise multiplication. Finally, the tracking feature ${\tilde{U}}_{t}^{3,4,5}$ at the current time step is updated by $U_{h_{t}}=O\left({U_{t,}}_{input}\right)$.

### 3.3. Objective Function

The total loss in this paper is boiled down to four components: focal loss $L_{cls}$ is used to make the bounding-box classification branch focus on more challenging classes during training, IoU loss $L_{reg}$ to implement bounding-box regression between predicted and ground-truth boxes, a pixelwise binary cross entropy (BCE) loss $L_{mask}$ for mask generation, and cross-entropy loss $L_{track}$ leveraged for the tracking branch, respectively, taking the form:

(10) $$L_{total}=L_{cls}+L_{reg}+L_{mask}+L_{track}$$

#### Tracking Loss

Supposing that *N* instances have been identified in previous frames, an agnostic instance *i* for the current frame could be assigned one of the identities when instance *i* belongs to one of the instances *N* in the previous frames; otherwise, a new ID is assigned by digit 0. Moreover, we denote each instance *i* and j∈[1,N] at time step *t* and t−1 as a new tracking feature vector $U_{track}^{t,i}$ and $U_{track}^{t-1,j}$, which are extracted from the tracking feature maps $U_{t}^{3,4,5}$ and $U_{t-1}^{3,4,5}$ generated by the TMU module in Section 3.3, respectively. We formulate instance tracking as an N+1 classification problem, and the probability $p_{i}\left(j\right)$ of assigning label *j* to the unseen instance *i* can be represented as:

(11) $$p_{i}\left(j\right)=\left\{\begin{array}{cc} \frac{\exp\left({\left(U_{track}^{t,i}\right)}^{T}U_{track}^{t-1,j}\right)}{1+\sum_{j=1}^{N}{\left(U_{track}^{t,i}\right)}^{T}U_{track}^{t-1,j}} & j\in [1,N]\\ \frac{1}{1+\sum_{j=1}^{N}{\left(U_{track}^{t,i}\right)}^{T}U_{track}^{t-1,j}} & j=0 \end{array}\right.$$

Eventually, $L_{track}$ for the tracking branch is generated by using the cross-entropy loss:

(12) $$L_{track}=-\sum_{i}\log\left(p_{i}\left(g_{i}\right)\right)$$

where $g_{i}$ indicates the ground truth identify for a new instance.

## 4. Experiments

### 4.1. Dataset

We empirically and thoroughly evaluate the performance of the HT-VIS on YouTube-VIS and the self-built UAV-VIS datasets. YouTube-VIS is a large-scale benchmark for the video instance segmentation task proposed in [15], which consists of 2883 YouTube videos with 40 categories at a frame rate of 30 FPS. It is split into train, valid, and test subsets with 2238, 302, and 338 video clips. As the ground truth labels are not officially disclosed for the validation dataset, this restricts the evaluation of our proposed method. Therefore, the training set of YouTube VIS is divided into two train–test splits in a proportion of 85–15% (1904 and 334 videos) following [21]. Moreover, for the sake of demonstrating the generalization ability and robustness of the proposed method, we take category characteristics into account and finally establish four different typical scenarios, which are Zoo, Street, Vehicle, and Sport. Among them, there are 549 and 108 video clips corresponding to 829 and 176 instances, respectively, in the Zoo scene. Furthermore, the training set and test set in the Street scene contain 284 videos with 542 instances and 44 videos with 79 instances, respectively. We extract 225 and 331 video clips from the Vehicle scene to construct two train–test splits composed of 542 and 79 instances. In the Sport scene, 153 video clips with 333 instances and 36 videos with 90 instances are made up of training sets and test sets, respectively.

**Figure 5:**
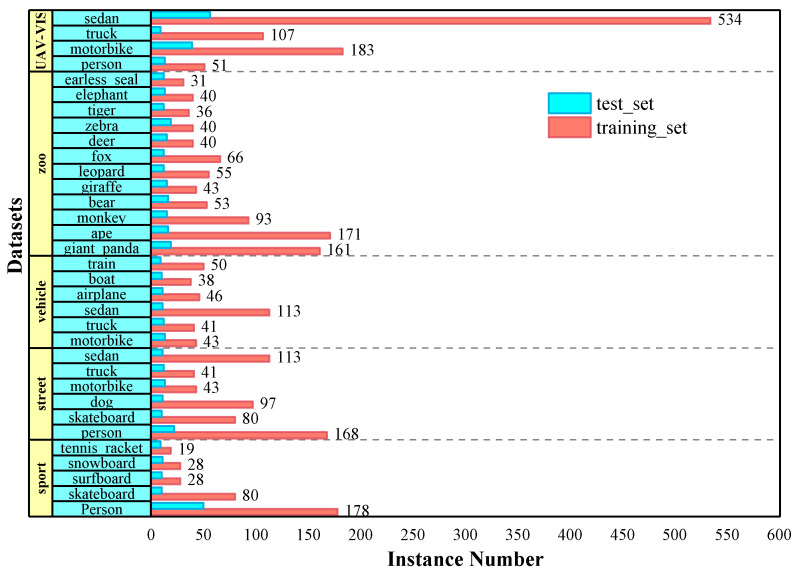
Distribution of different categories included in four typical scenario datasets and UAV-VIS dataset.

The UAV-VIS dataset for the video instance segmentation task is derived from (1) the publicly available visible-thermal imaging UAV large-scale dataset VIUAV and (2) self-built video from the UAV perspective. The self-constructed dataset was collected by a DJI Mini 3 drone in Kunming city. It contains 163 video sequences with 6397 frames. Further, 290 videos with 11,584 frames are randomly selected from the VIUAV dataset and then integrated with the collected 163 videos to build the UAV-VIS dataset, containing 453 videos and 17,981 frames. In addition, a semi-automatic labeling tool called X-AnyLabeling is used to generate fine pixel-level mask annotations on the instances of four typical categories, including person, sedan, motorbike, and truck. All the labeled videos with a resolution of 1920 ×1080 were divided into training and validation sets in the ratio of 85%:15%. The distribution of instances for the four scene datasets and the UAV-VIS dataset are shown in Figure 5.

### 4.2. Implementation Details

#### 4.2.1. Training

All experiments in this paper are implemented with an NVIDIA RTX 2080Ti GPU and the PyTorch 1.1.0 framework under CUDA 11.2 and cuDNN 8.0.5. The input image sizes are set to 640 × 360 pixels before being fed into the network for memory efficiency, and the batch size is set to 6; an SGD optimizer is employed with the initial learning rate of $5\times 10^{-4}$ and a weight decay of $1\times 10^{-4}$ by step learning strategy $lr=base\_lr\times 0.1^{\frac{epoch}{\mathrm{int}\left(num\_epoch/epoch\_step\right)}}$ at the 8th epoch and 10th epoch, respectively, to ensure faster convergence.

#### 4.2.2. Inference

In the inference step, we set the NMS threshold to 0.3. Compared to SipMask, which merely uses the current frame to segment instances at each time step during inference, a reference frame is added and fed into the TMU module with the current ones in our method to ensure the instances’ tracking performance.

### 4.3. Evaluation Metrics

Segmentation accuracy is used for the evaluation metric, which is evaluated by average precision (AP) with the video intersection-over-union (IoU) thresholds at 0.50 and 0.75 and average recall (AR). Since each instance is represented by a sequence of different masks, following Mask Track-RCNN, we compute the IoU between a ground truth instance and a hypothesis instance ${\tilde{m}}_{\tilde{a}\ldots \tilde{b}}^{\tilde{j}}$. First, we extend *a* and $\tilde{a}$ to 1 and *b* and $\tilde{b}$ to *T* by padding empty masks if there are no instances of a frame in the video sequence. The IOU is computed as:

(13) $$IOU\left(i,j\right)=\frac{\sum_{t=1}^{T}m_{t}^{i}\cap {\tilde{m}}_{t}^{j}\;\;\;\;\;\;}{\sum_{t=1}^{T}m_{t}^{i}\cup {\tilde{m}}_{t}^{j}\;\;\;\;\;\;}$$

Then, average precision (AP) and average recall (AR) are updated as:

(14) $$AP=\frac{TP}{TP+FP}\times 100\%$$

### 4.4. Performance

#### 4.4.1. Effectiveness of the Hierarchical Offset Compensation

As shown in Table 2, the experimental results demonstrate that the segmentation accuracy on the four scenario datasets is notably improved compared to the results obtained by the baseline, which exhibits high generalization and performance in both the SHOC and PHOC modules. Specifically, the model using the SHOC module achieves notable improvement (+1.8%, +1.7%. +3.2%, and +3.2% in AP) on the Zoo, Vehicle, Street, and Sport validation datasets, respectively, while leveraging the PHOC module outperforms the baseline SipMask by 2.0%, 2.1%, 2.8%, and 2.8% in AP, respectively. This indicates that the segmentation performance can be effectively improved by our proposed HOC module.

**Figure 6:**
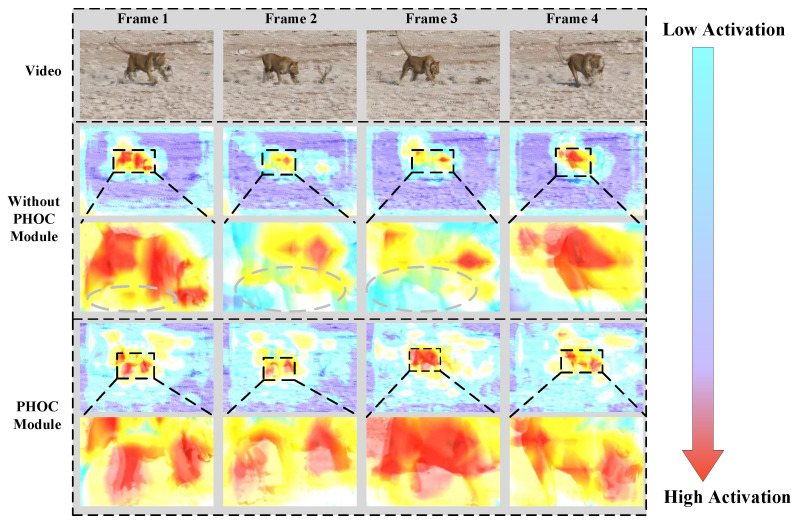
Quantitative comparison of AP across categories on the Zoo dataset (the transition from cyan to red in the color bar represents a change from low to high activation).

For better demonstration, we compared the visual heatmap results of our proposed model with and without the PHOC module, as depicted in Figure 6. Grad-CAM is used to calculate the last convolution layer of the PHOC module to generate attention maps, where the black dashed box indicates the detail regions while the gray dashed box highlights the discriminative feature areas. As shown in Figure 6, in the first column, the model without the PHOC module produced a heatmap focused on the foreground target body part while ignoring limb movements. The reason for this phenomenon is that the baseline model is incapable of capturing the main positional movements of the topological target, which is essential for compensating for spatial motion offsets across frames, while our proposed PHOC is capable of generating attention maps effectively to highlight the body parts and limb movements with large geometric variations for the target. In the fourth column, we observed that the critical head feature is captured by our proposed model with the PHOC efficiently compared to the baseline. This manifests the effectiveness of the PHOC module in improving the target motion performance through compensating offset features between adjacent frames.

#### 4.4.2. Effect of Temporal Memory

**Figure 7:**
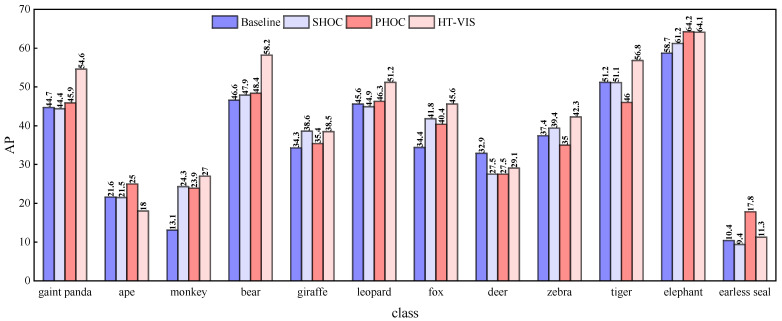
Quantitative comparison of AP across categories on Zoo dataset.

Optimal experimental results are obtained on four typical scenario datasets by employing the SHOC and PHOC modules, respectively, where the PHOC module outperformed SHOC on the Zoo and Vehicle datasets, while the SHOC module exhibited superior results on the Street and Sport scenes. To verify the effectiveness of the proposed TMU model on two kinds of HOC, of which the best one is selected as our benchmark, the experimental results are illustrated in Table 3. As can be observed, the incorporation of SHOC/PHOC and TMU further achieves the overall improvement of 3.5%, 0.1%, and 0.6% in terms of AP on the three scenario datasets of Zoo, Vehicle, and Street, respectively, demonstrating that PHOC and TMU are complementary to each other for learning the hierarchical motion offsets of irregular instances and tracking. In addition, we also analyze the effectiveness of TMU for each category on the Zoo dataset, as depicted in Figure 7. Note that HT-VIS achieves a significant performance over the baseline and SHOC/PHOC module in the majority of categories. In particular, in terms of the giant panda presenting a relatively large number of instances, PHOC bolsters the performance from 44.7% AP to 45.9% AP, and TMU ameliorates the PHOC by 8.7% (45.9% vs. 54.6%). Furthermore, the HT-VIS obtains 27% AP of the monkey, which is 13.9% higher than the SipMask baseline by a large margin. This substantiates the effectiveness of TMU in improving the tracking accuracy.

There exists a clear tendency that the performance after embedding the TMU module on the SHOC increases by 1.3% compared to the SipMask baseline while declining by 1.9% versus the SHOC module on the Sport dataset. One potential reason for this is that moving targets, such as people on skateboards in motion scenarios, exhibit more than topological deformation and inter-occlusion, which is also presented with prominent behavioral uncertainty. Our HOC module is capable of handling irregularly moving targets in the time series as well as learning the hierarchical motion features. However, when the movement direction and deformation of the target in the next moment are strictly agnostic, which will hinder the TMU module from memorizing the previous moments for further understanding and learning, the model fails to contact the target’s motion behavior well in the time series.

Table 4 shows the ablation experiments of different modules of HT-VIS on the UAV-VIS dataset. After embedding the SHOC and PHOC module, the benchmark model SipMask improves the segmentation accuracy from 35.3% by 1.4% and 1.3% to 36.7% and 36.6%, respectively. On this basis, the model accuracy after embedding the TMU module reaches 37.1% and 37.4%, which is 1.8% and 2.1% higher than that of SipMask, respectively, verifying the robustness and effectiveness of HT-VIS.

#### 4.4.3. Ablation Study

To investigate the contribution of the HOC module in sequential and parallel connection to reconstruct layer $M_{l}$ for the current frame, we conduct extensive ablation experiments on the same backbone (ResNet-50) for a fair comparison. Table 5 shows that the proposed SHOC and PHOC modules exhibit significant improvements in accuracy compared to the baseline for each layer of the reconstructed FPN. Specifically, the SHOC module achieves the highest accuracy of 40.2% at layer $M_{5}$ while the PHOC module obtains the optimal accuracy of 39.6% at layer $M_{2}$. This demonstrates that the proposed HOC module helps the encoder learn motion offset better for the inter-frame instance.

Then, we conducted a comparative experiment to evaluate the effectiveness of integrating TMU into each layer of the HOC module. As illustrated in Table 6, the results showcase that the SHOC+TMU and PHOC+TMU models exhibit enhanced segmentation accuracy. When embedding the TMU module into the SHOC model, the best accuracy achieves an AP of 41.2% at layer $M_{5}$, which increased by 1%, whereas the PHOC+TMU obtains the segmentation accuracy of 41.4% at layer $M_{3}$ with a 3.5% improvement compared to the PHOC model. It is noteworthy that the optimal accuracy of the PHOC and PHOC+TMU models are at layers $M_{2}$ and $M_{3}$, where the feature map size is 6×10 and 12×20, respectively. This result is attributed to the central position of $M_{3}$ in the FPN, which is moderate and allowed to capture high-level semantic features and low-level granularity information simultaneously. In addition, features at different scales are effectively extracted by each layer of various dilation filters to further enhance the representation of the $M_{3}$ layer features. Consequently, following the PHOC module at the $M_{3}$ layer and subsequently adding the TMU to learn and memorize the state of inter-frame instances at each time step could effectively improve the tracking performance.

In addition, we explore the effect of adding the TMU tracking module for inference performance. As shown in Table 7, the results demonstrate that adding the TMU module improves the tracking performance significantly compared to the case where it is not added. Specifically, the segmentation accuracy at the $M_{3}$ layer is only 28.5% when the PHOC+TMU is tested without the TMU module, while the tracking accuracy improves to 41.4% after adding the TMU module. This result further validates the effectiveness of the TMU module in object tracking and indicates that it can provide an important effect in high-precision segmentation.

### 4.5. Error Analysis

To further demonstrate the fact that the proposed HT-VIS indeed shows the strength of spatial segmentation and temporal correlation for calculating mAP, the error diagnosis toolbox developed by [40] is adopted for the video instance segmentation task. The errors generated by the models can be briefly divided into false positives (FPs) and false negatives (FNs). However, it is necessary to further distinguish a set of error types since these two ones obfuscate the referenced factors during evaluation. First, $IoU_{\max}$ is defined by the best-matched mask sequence IoU of a false positive to the ground truth (GT) for the specified category; the background threshold $\theta_{b}$ and the foreground threshold $\theta_{temp}$ are set to 0.1 and 0.5, respectively. $Y_{match}$ denotes the number of frames whose mask $IoU_{t}$ are higher than the mask sequence IoU, where $IoU_{t}$ is defined as Equation (13). Then, $overlap_{temp}$, denoted by the tracking quality for predictions, is the ratio of $Y_{match}$ to the temporal union $\left\{a,\ldots b\right\}\cup \left\{\tilde{a},\ldots \tilde{b}\right\}$, which is computed as Equation (15):

(15) $$overlap_{temp}=\frac{Y_{match}}{\left\{a,\ldots b\right\}\cup \left\{\tilde{a},\ldots \tilde{b}\right\}}$$

The temporal association threshold $\theta_{temp}$ is set to 0.7. Finally, seven error types and definitions are developed by three variables of category, $IoU_{\max}$, and $overlap_{temp}$, as described in Table 8. In addition, the variation ΔAP is employed to reflect intuitively the extent to which individual error types for each category hold back the performance of the model.

**Figure 8:**
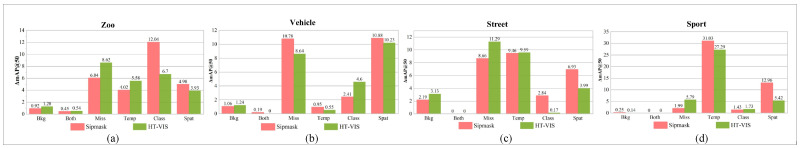
Error distribution of SipMask and HT-VIS on four datasets.

**Figure 9:**
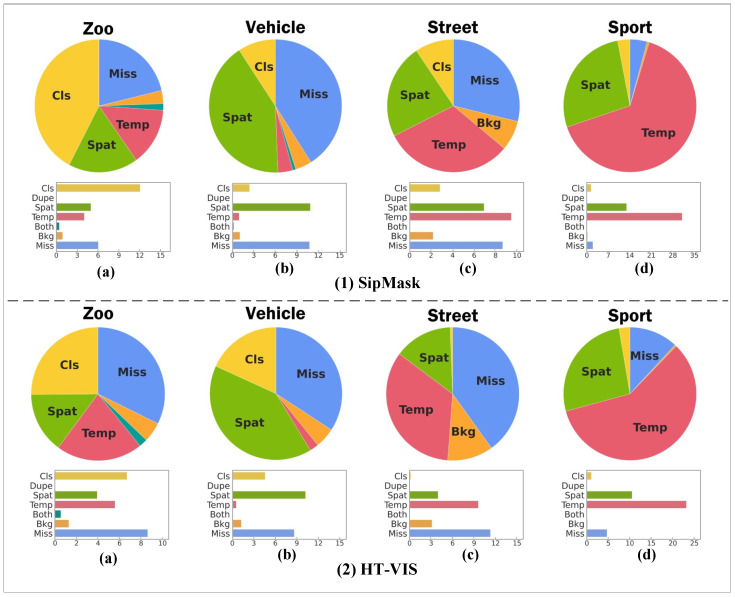
Error comparison of SipMask and HT-VIS on four datasets.

Figure 8 and Figure 9 illustrate the relative error distributions and absolute comparison results for SipMask and HT-VIS on the four scenario datasets, respectively. From Figure 9a,c, it can be seen that the spatial segmentation error weight of HT-VIS is less than that of SipMask without spatial feature compensation, demonstrating the effectiveness of HT-VIS in reconstructing current frame features from spatial motion offsets. Together with Figure 8a,c, we can observe that, compared to SipMask, HT-VIS has better target classification and instance-level segmentation, verifying that our proposed method is able to improve the segmentation results by reducing the spatial error weight through the hierarchical offset compensation module. In Figure 9b,d, it is intuitively obvious that the temporal error and spat error weights of HT-VIS decline significantly compared to SipMask. In association with Figure 8b,d, HT-VIS achieves the continuous tracking and segmentation of moving targets, indicating that the temporal memory update module in this paper is critical for establishing temporal association.

#### 4.5.1. Visualisation

**Figure 10:**
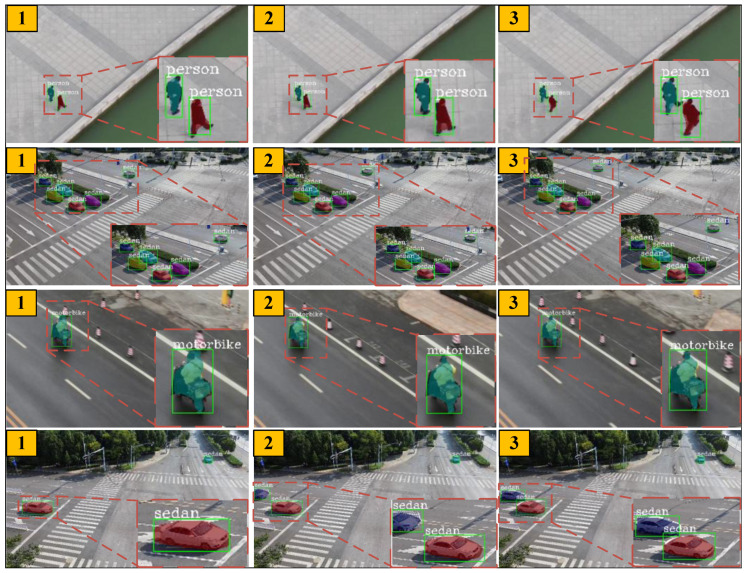
Visualization of HT-VIS on the self-built UAV-VIS dataset. (The numbers in the yellow regions indicate frame samples in the video sequence. The red dashed box indicates the amplified area).

The self-built UAV-VIS dataset. Figure 10 visualizes some examples of our HT-VIS on the UAV-VIS dataset. The first three rows show where HT-VIS consistently and accurately segments and tracks individual instances of persons, sedans, and motorbikes in motion. Furthermore, in the last row, when a new car instance appears, HT-VIS successfully assigns it a distinct ID while maintaining uninterrupted tracking of the original target, validating the robustness and effectiveness of the proposed methodology.

**Figure 11:**
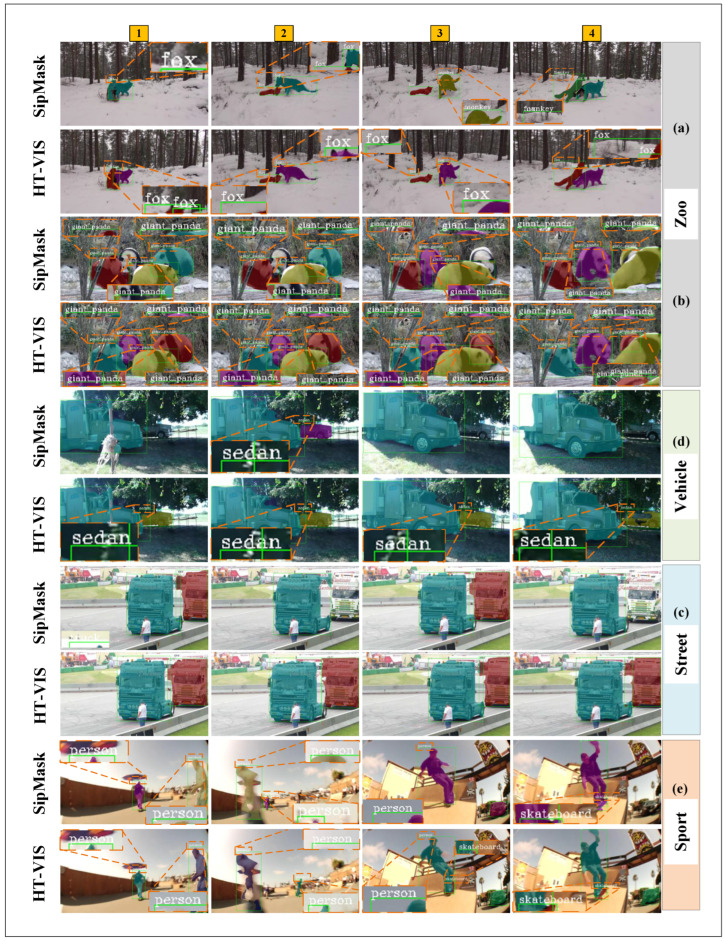
Visualization of HT-VIS and SipMask on the four UAV scenario test datasets. (**a**), (**b**), (**c**) and (**d**) represent the visualization results of the Zoo, Street, Vehicle, and Sport scenes, respectively. In each scene, the first line shows the masks of the four frame samples predicted by SipMask, and the second row shows the predicted masks output by HT-VIS. The numbers in the yellow regions indicate frame samples in the video sequence. The red dashed box indicates the amplified area.

Four typical scenario datasets constructed by YouTube-VIS-2019. To qualitatively evaluate the proposed method, Figure 11 visualizes and compares the segmentation results with the SipMask benchmark of some examples from four typical UAV scenario datasets, including Zoo, Street, Vehicle, and Sport. Frames are sampled at certain important moments (e.g., multi-object occlusion and deformation). The following can be observed: (1) In video (a), SipMask segments two foxes into one target mask in the first frame, and although accurate segmentation is achieved in the second frame, it still shows instance identity tracking error and category detection error in the next two frames. In contrast, our model shows good robustness in detection, segmentation, and tracking. (2) Video (b) shows the comparative effect of segmentation on multiple objects. It can be seen that, when segmenting the panda in the upper right corner, the benchmark model has no segmentation on the second panda in the first two frames and incorrect tracking in the second two frames, as shown by the two pandas having the same color mask. (3) In video (c), the benchmark model has difficulty segmenting and tracking the car due to the occlusion of the tree; our method achieves more accurate segmentation results. (4) As shown in video (d), the right truck was unsteadily segmented and tracked by the benchmark model, and the tracking result was lost in the third frame. (5) In video (e), the instance scale and appearance of the person on the skateboard change considerably due to the rapid motion, and in the second frame it was incorrectly tracked as a passerby because the benchmark model fails to learn effective offset features and temporal memory. By analyzing the above visualization results with the mechanisms of the proposed hierarchical offset compensation and temporal memory, it can be illustrated that our model can detect, segment, and track the same target instance more accurately for some typical problems in video tasks such as topological deformation, scale transformation, and occlusion of multi-instance in motion.

#### 4.5.2. Comparing with Existing Methods

To demonstrate the comprehensive performance of the proposed model, we compare it with approaches proposed in [16,19,21,22] on the developed four UAV scenario datasets. Note that we use ResNet-50 as the backbone, and the experimental results are shown in Table 9. As can be seen, our method outperforms other video instance segmentations by a significant margin with respect to most of the evaluation metrics. Specifically, the segmentation accuracy of our HT-VIS achieves the best segmentation results, with AP of 41.4%, 41.1%, 38.5%, and 18.2%, respectively, on the four UAV scenario datasets. Moreover, a comprehensive comparison with state-of-the-art VIS approaches is conducted on the pixel-wise annotated UAV-VIS dataset, as displayed in Table 10. It is evident that the proposed HT-VIS with the PHOC module and SHOC module achieves an mAP of 37.4% and 37.1%, respectively, which separately outperforms the baseline model SipMask by 2.1% and 1.8%. Note that the average segmentation accuracy of HT-VIS with PHOC is 1.2% higher than that of the latest CNN-based VIS method CrossVIS. In the transformer-based VIS methods, VISAGE achieves the highest segmentation accuracy of 46.7%, while the SeqFormer model obtains a lowest mAP of 39.4% with parameters of 220 M. The HT-VIS makes a trade-off between the accuracy of 37.4% and parameter of 35 M. These experiments fully demonstrate the effectiveness and superior performance in the constructed UAV-VIS datasets. These experiments fully demonstrate that our method achieves superior segmentation performance in all constructed UAV scenario datasets.

**Figure 12:**
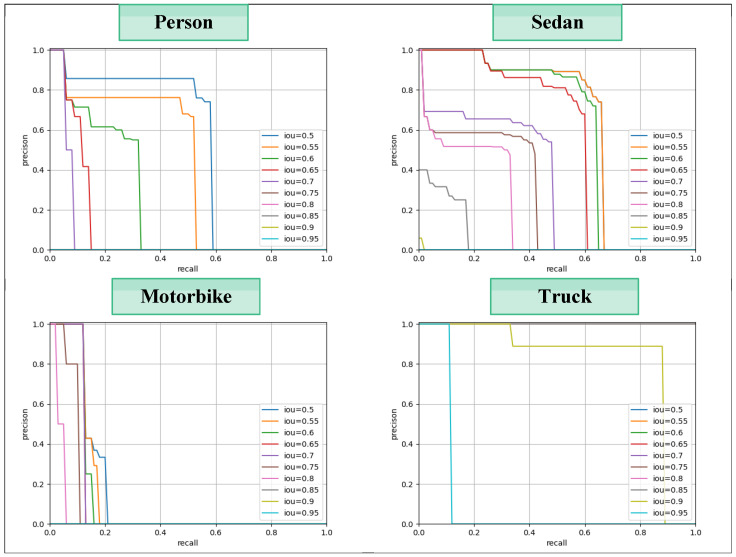
PR curves for different categories at various thresholds of the HT-VIS model.

The precision–recall (P-R) curves in Figure 12 comprehensively evaluate the HT-VIS model’s detection performance across four categories (person, sedan, motorbike, and truck) under varying IoU thresholds. For the person category, there is a significant decrease in precision as the IoU threshold rises, indicating the model struggles to maintain high precision for strict overlap requirements when detecting people. The sedan category shows strong performance, maintaining stable precision over a wide range of recalls. Detection precision for the motorbike category decreases rapidly with increasing recall, which is mainly attributed to the small size of the objects and frequent occlusions. Notably, the model amplifies the sensitivity of the IoU to minor localization errors due to the large target size percentage of the image; the truck category shows measurable detection performance only at IoU≥0.9. By analyzing the PR curves for the four categories, the effectiveness and limitations of the model for segmenting the different categories could be effectively assessed.

### 4.6. Edge Device of Deployment

To validate the effectiveness of the proposed HT-VIS in different experimental setups, an embedded test platform was developed to perform experiments. The platform employs the Ubuntu 18.04 operating system and an NVIDIA Jetson Nano Developer Kit-B01 (“NVIDIA Corporation, Santa Clara, CA, USA”) was selected to deploy the HT-VIS model. The key technical specifications are detailed in Table 11. Before model deployment on the edge computing device Jetson Nano Kit-B01, we established the operating system configuration and constructed the runtime environment. To guarantee system integrity and hardware compatibility, the Ubuntu 18.04 LTS operating system image with an integrated visual desktop environment was initially flashed onto the device’s storage medium using the Balena Etcher tool. Following this system setup, we sequentially installed the Python 3.8 programming environment and essential compilation components, specifically the NVIDIA CUDA toolkit, PyTorch deep learning framework, and NumPy scientific computing library. The software environment on the edge device was rigorously verified to precisely match the experimental conditions outlined in Section 4.2.1, thereby ensuring the complete reproducibility of both model performance and experimental results post-deployment. The segmentation accuracy of the HT-VIS model for inference on edge devices remains consistent with that on GPUs, as shown in Table 10. HT-VIS with SHOC and PHOC modules achieves segmentation accuracies of 59.0% and 58.9% in mAP, respectively. FPS is used as an additional evaluation metric for the effectiveness of HT-VIS deployment to the NVIDIA Jetson Nano Developer Kit-B01 edge device. Experiments demonstrate that the inference time for a single frame is 2 s.

**Figure 13:**
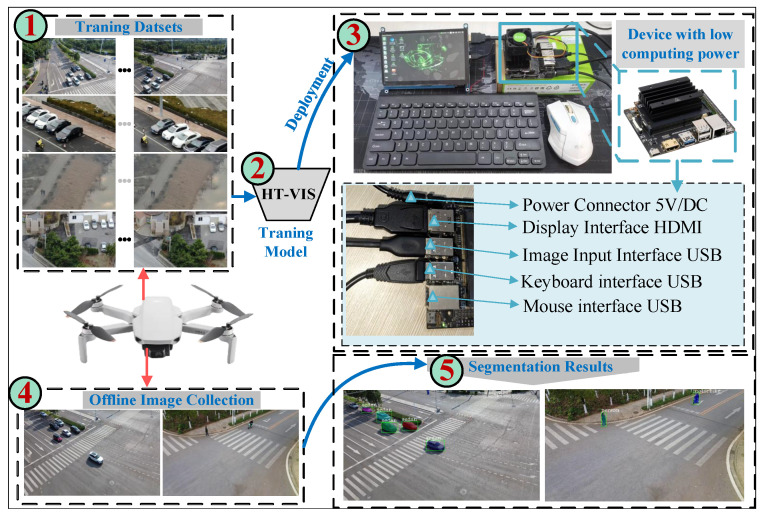
Diagram of model deployment. (Red arrows indicate data collection by UAVs, dark blue arrows and numbers indicate deployment steps).

The deployment process is illustrated in Figure 13. The dataset collected by UAV is first trained through the proposed HT-VIS framework to obtain the model weight and is then deployed onto the NVIDIA Jetson Nano Developer Kit-B01. Subsequently, the deployed model is used to evaluate the test data to generate segmentation results. Based on the quality of the output masks, it can be proven that our model can provide effective segmentation performance for multi-scale topological deformation targets.

## 5. Conclusions

In this study, we propose an efficient HT-VIS framework for video instance segmentation in UAV patrol and inspection tasks. Firstly, to effectively handle the topological deformations of multiple instances within the same category, we design a hierarchical offset compensation module with sequential and parallel connections to learn sufficient geometric information about the instances. Secondly, to address the issue of tracking loss that occurs during instance motion, we develop a spatio-temporal memory update strategy, which facilitates the establishment of spatio-temporal dynamic contextual correlations between individual frames, thereby enhancing the model’s ability to track instances over time. Furthermore, to evaluate the effectiveness of the proposed HT-VIS framework, we construct an episodic dataset that includes Zoo, Street, Traffic, and Sport scenarios derived from the YouTube-VIS dataset to simulate the different typical inspection tasks from UAV perspectives. Moreover, a UAV aerial dataset named UAV-VIS is built in this paper for a comprehensive assessment of the proposed HT-VIS framework. Finally, an NVIDIA Jetson Nano Developer Kit-B01 device with low computing power is used to deploy the trained HT-VIS model weights for the segmentation of target instances. The extensive experiments show the competitive segmentation accuracy of the HT-VIS, demonstrating the effectiveness and robustness in UAV aerial image segmentation and tracking tasks.

## 6. Discussion

Although this study focuses on the validation of the effectiveness of the HOC and the TMU module for video instance segmentation in a normal scenario from the UAV perspective, the architecture is designed to have a certain potential noise robustness. HT-VIS helps to extract target features from noise by learning the continuous frame target motion deviation and spatio-temporal modeling mechanism, but these advantages still need further experimental validation. In our future work, we plan to quantify the attenuation of model performance metrics under different noise intensities by adding Gaussian noise and motion blur to the samples; secondly, we will collect datasets from real harsh environments, such as foggy days and low-light conditions, to validate the model’s robustness in complex real-world scenarios.

## 7. Limitations and Future Work

Although the proposed HT-VIS has made progress, there are still limitations. The designed temporal memory update module could model local interframe temporal features using adjacent frames based on the ConvLSTM mechanism. However, the TMU module is limited in capturing the clip-level features because the video sequences are continuously dense and the target is dynamically changed, failing to accurately comprehend the global contextual information of the instances and to perceive the motion situation. Therefore, in future work, we will introduce a lightweight Transformer architecture to model more temporal features with lower computational resources and number of parameters to improve segmentation and tracking accuracy.

## Figures and Tables

**Table 1:** Definition of symbolic variables.

Notations	Defination
*l*	The l−th layer of FPN
*n*	The number of spatial motion offset (SMO) mechanisms
*r*	dilation factor
$f_{l,0}$	Sampling center point with offset of 0
$f_{l,r}$	Predefined offset sample location in the regular convolution
ω(·)	The weights of the deformable convolution kernel
$\Delta f_{l,r}^{t-1\to t}$	The learnable offset from time step t−1 to *t*
${F_{l,in}}^{t-1\to t}$	Input feature map for HOC module
$F_{l,out}^{t-1\to t}$	The output feature map of the deformable convolution on the sample location $f_{l,0}$ from the time step t−1 to *t*
$\Theta_{r}$	A sample point set of r×r a grid
$\Delta f_{l,n}^{t-1\to t}$	The n−th offset feature of the SMO mechanism from time step t−1 to *t*
$D_{r}^{p}\left(\cdot \right)$	The deformable convolution operation with k×k×256 represented by Equation (1)
$\Lambda_{r}^{p}$	Standard convolution filter with padding *p* and dilation rate *r* in the SMO mechanism
$\varphi_{3\times 3}^{s}\left(\cdot \right)$	A 3×3 standard convolution with a stride of *s*

**Table 2:** HOC module effects.

Scene	Model	AP	AP@50	AP@75	AR@1	AR@10	AR@s	AR@m	AR@l
Zoo	Baseline	35.9	58.3	42.1	35.6	44.5	12.0	51.4	52.7
	SHOC	37.7 (+1.8)	58.6 (+0.3)	45.4 (+3.3)	36.5 (+0.9)	45.1 (+0.6)	13.9 (+1.9)	53.5 (+2.1)	50.2
	PHOC	37.9 (+2.0)	59.1 (+0.8)	44.9 (+2.8)	37.0 (+1.4)	45.2 (+0.7)	12.5 (+0.5)	52.1 (+0.7)	53.0 (+0.3)
Vehicle	Baseline	38.9	66.5	37.6	41.4	45.9	38.9	31.9	54.8
	SHOC	40.6 (+1.7)	65.0	39.6 (+2.0)	42.9 (+1.5)	46.3 (+0.4)	29.4	39.2 (+8.7)	56.6 (+1.8)
	PHOC	41.0 (+2.1)	65.5	44.7 (+7.1)	44.4 (+3.0)	47.3 (+1.4)	36.1	40.6 (+8.7)	57.0 (+2.2)
Street	Baseline	33.5	59.0	35.9	35.4	40.2	12.6	22.1	57.0
	SHOC	36.7 (+3.2)	63.1 (+4.1)	37.4 (+1.5)	39.5 (+4.1)	44.1 (+3.9)	15.6 (3.0)	32.8 (+10.7)	63.9 (+6.9)
	PHOC	36.3 (+2.8)	65.6 (+6.6)	35.7	39.9 (+4.5)	45.4 (+5.2)	19.1 (+6.5)	30.8 (+8.7)	60.3 (+3.3)
Sport	Baseline	15.0	34.3	12.7	16.5	17.9	16.1	29.5	32.5
	SHOC	18.2 (+3.2)	46.1 (+11.8)	11.0	18.3 (+1.8)	19.6 (+1.7)	17.9 (+1.8)	44.8 (+25.3)	21.7
	PHOC	17.8 (+2.8)	47.2 (+12.9)	11.6	17.7 (+1.2)	19.0 (+1.1)	18.0 (+1.9)	30.2 (+0.7)	24.2

**Table 3:** TMU module effects.

Scene	Model	AP	AP@50	AP@75	AR@1	AR@10	AR@s	AR@m	AR@l
Zoo	PHOC	37.9	59.1	44.9	37.0	45.2	12.5	52.1	53.0
	+TMU	41.4 (+3.5)	61.9 (+2.8)	46.5 (+1.6)	39.1 (+2.1)	48.3 (+3.1)	15.6 (+3.1)	54.6 (+2.5)	54.9 (+1.9)
Vehicle	PHOC	41.0	65.5	44.7	44.4	47.3	36.1	40.6	57.0
	+TMU	41.1 (+0.1)	65.9 (+0.4)	42.5	44.7 (+0.3)	47.4 (+0.1)	37.8 (+1.7)	30.3	57.9 (+0.9)
Street	SHOC	36.7	63.1	37.4	39.5	44.1	15.6	32.8	63.9
	+TMU	38.5 (+0.6)	65.3	40.3 (+2.8)	40.3 (+2.8)	45.7 (+0.7)	16.4	36.4 (+1.9)	61.7 (+4.4)
Sport	SHOC	18.2	46.1	11.0	18.3	19.6	17.9	44.8	21.7
	+TMU	16.3	47.6	10.1	15.1	17.6	18.0	37.0	20.8

**Table 4:** Ablation experiments on the UAV-VIS dataset (✘: disabled; ✔: enabled).

Module			mAP	mAP@50	mAP@75	AR@1	AR@10
**SHOC**	**PHOC**	**TMU**					
✘	✘	✘	35.3	58.4	33.9	26.9	39.1
✔	✘	✘	36.7	57.5	35.5	28.0	39.9
✘	✔	✘	35.9	58.2	34.5	27.2	40.0
✔	✘	✔	37.1	59.0	37.8	28.4	41.9
✘	✔	✔	37.4	58.9	39.9	29.6	42.2

**Table 5:** Ablation experiments for the layer of FPN to be reconstructed by HOC module (✘: disabled).

Model	Layer	AP	AP@50	AP@75	AR@1	AR@10	AR@s	AR@m	AR@l
baseline	✘	35.9	58.3	42.1	35.6	44.5	12.0	51.4	52.7
SHOC	1	38.6	58.8	42.1	37.2	46.1	15.8	54.9	50.1
	2	39.7	60.2	44.1	37.0	45.7	16.4	51.8	52.2
	3	37.7	58.6	45.4	36.5	45.1	13.9	53.5	50.2
	4	39.8	59.6	44.6	36.9	46.5	15.9	50.9	53.7
	5	40.2	59.5	56.3	37.4	47.0	16.4	52.4	54.8
PHOC	1	37.1	57.1	41.4	35.0	42.8	13.7	49.8	47.1
	2	39.6	62.3	43.0	37.4	45.8	15.8	52.2	49.3
	3	37.9	59.1	44.9	37.0	45.2	12.5	52.1	53.0
	4	39.6	60.1	44.9	38.1	47.1	19.6	51.9	53.1
	5	39.5	58.5	45.7	37.1	46.9	19.1	51.9	53.6

**Table 6:** Ablation experiments on each layer of HOC module of embedded TMU.

Layer	AP	AP@50	AP@75	AR@1	AR@10	AR@s	AR@m	AR@l	AP	AP@50	AP@75	AR@1	AR@10	AR@s	AR@m	AR@l
	**SHOC+TMU**								**PHOC+TMU**							
1	39.7	59.8	43.0	38.3	46.6	13.9	50.6	54.6	38.9	60.6	41.6	38.2	46.0	13.6	48.2	53.6
2	40.7	63.0	45.3	39.1	47.3	18.0	49.9	55.1	39.8	59.6	45.2	38.4	47.3	16.5	50.9	54.2
3	40.8	60.6	47.0	38.6	47.1	16.9	51.2	53.2	41.4	61.9	46.5	39.1	48.3	15.6	54.6	54.9
4	40.5	61.1	47.1	38.6	47.0	15.8	53.9	52.2	40.7	60.5	45.9	37.9	46.5	14.7	51.0	53.3
5	41.2	61.0	46.9	37.8	47.5	19.2	53.2	54.4	39.7	59.9	44.5	38.2	46.1	19.0	50.9	52.8

**Table 7:** Ablation experiments for tracking during test.

Layer	AP	AP@50	AR@1	AR@10	AP	AP@50	AR@1	AR@10	AP	AP@50	AR@1	AR@10	AP	AP@50	AR@1	AR@10
	**SHOC+TMU**								**PHOC+TMU**							
	**w/o Tracking**				**with Tracking**				**w/o Tracking**				**with Tracking**			
1	26.4	42.8	25.5	32.8	39.7	59.8	38.3	46.6	25.8	44.7	25.6	30.6	38.9	60.6	38.2	46.0
2	30.6	50.8	30.8	37.2	40.7	63.0	39.1	47.3	29.1	47.7	30.4	36.1	39.8	59.6	38.4	47.3
3	28.7	50.5	26.7	34.9	40.8	60.6	38.6	47.1	28.5	47.1	27.8	35.4	41.4	61.9	39.1	48.3
4	29.0	46.8	28.1	36.1	40.5	61.1	38.6	47.0	26.4	46.6	27.5	34.1	40.7	60.5	37.9	46.5
5	28.1	44.9	27.1	33.3	41.2	61.0	37.8	47.5	29.5	47.5	29.2	36.6	39.7	59.9	38.2	46.1

**Table 8:** Error types and definitions.

Error Type		Error Definition
False Positive (FP)	background error (Bkg)	${\mathrm{IoU}}_{\max}<\theta_{b}$ and ${\mathrm{overlap}}_{\mathrm{temp}}<\theta_{\mathrm{temp}}$ for all foreground categories with ground truth
	classification error (Cls)	${\mathrm{IoU}}_{\max}\geq \theta_{f}$ and ${\mathrm{overlap}}_{\mathrm{temp}}\geq \theta_{\mathrm{temp}}$ with incorrect category
	both classifications and location error (Both)	$\theta_{f}>{\mathrm{IoU}}_{\max}\geq \theta_{b}$ and $0<{\mathrm{overlap}}_{\mathrm{temp}}<1$ with incorrect category
	duplication error (Dup)	${\mathrm{IoU}}_{\max}\geq \theta_{f}$ and ${\mathrm{overlap}}_{\mathrm{temp}}\geq \theta_{\mathrm{temp}}$ with correct category but another higher sequence IoU has matched corresponding GT
	spatial segmentation error (Spat)	$\theta_{f}>{\mathrm{IoU}}_{\max}\geq \theta_{b}$ and ${\mathrm{overlap}}_{\mathrm{temp}}\geq \theta_{\mathrm{temp}}$ with correct category
	temporal association error (Temp)	$\theta_{f}>{\mathrm{IoU}}_{\max}\geq \theta_{b}$ and ${\mathrm{overlap}}_{\mathrm{temp}}\leq \theta_{\mathrm{temp}}$ with correct category
False Negative (FN)	missed ground truth error (Miss)	All GT are not detected from foreground and not covered by any error.

**Table 9:** Comparison of the state-of-the-art methods on four typical scenarios constructed from the YouTube-VIS-2019 dataset.

Scene	Model	AP	AP@50	AP@75	AR@1	AR@10	AR@s	AR@m	AR@l
Zoo	SipMask	35.9	58.3	42.1	35.6	44.5	12.0	51.4	52.7
	STMask	36.9	52.5	41.5	34.2	41.5	13.5	45.9	43.0
	YolactEdge	31.5	58.9	41.5	-	-	-	-	-
	CrossVIS	37.5	56.5	43.3	36.6	47.3	15.0	52.4	53.2
	HT-VIS	41.4	61.9	46.5	39.1	48.3	15.6	54.6	54.9
Vehicle	SipMask	38.9	66.5	37.6	41.4	45.9	38.9	31.9	54.8
	STMask	27.8	44.4	27.6	31.2	33.0	18.3	13.6	44.5
	YolactEdge	32.4	59.5	34.0	-	-	-	-	-
	CrossVIS	39.1	61.7	38.9	40.8	45.1	38.3	37.2	53.1
	HT-VIS	41.1	65.9	42.5	44.7	47.4	37.8	30.3	57.9
Street	SipMask	33.5	59.0	35.9	35.4	40.2	12.3	22.1	57.0
	STMask	35.7	62.7	34.5	38.7	42.6	13.1	32.5	47.2
	YolactEdge	31.2	57.1	35.4	-	-	-	-	-
	CrossVIS	38.6	62.1	41.2	38.7	45.5	30.7	32.1	57.0
	HT-VIS	38.2	65.3	40.3	41.4	45.7	16.4	36.4	61.7
Sport	SipMask	15.0	34.3	12.7	16.5	17.9	16.1	29.5	32.5
	STMask	10.9	33.7	8.1	12.3	14.2	13.9	29.5	11.7
	YolactEdge	16.9	40.2	11.2	-	-	-	-	-
	CrossVIS	14.4	35.7	11.6	17.5	19.4	18.2	30.2	31.7
	HT-VIS	18.2	46.1	11.0	18.3	19.6	17.9	44.8	21.7

**Table 10:** Comparison of the state-of-the-art CNN-based VIS methods on the self-built UAV-VIS dataset.

Model		Year	mAP@50	mAP	FPS	Para.(M)
CNN-based Methods	SipMask [16]	2020	58.4	35.3	24	33
	CrossVIS [22]	2021	57.9	36.2	28	37
	HT-VIS(SHOC)	-	59.0	37.1	12	36
	HT-VIS(PHOC)	-	58.9	37.4	14	35
transformer-based Methods	SeqFormer [28]	2022	60.4	39.4		220
	MinVIS [34]	2022	65.8	44.2	-	-
	CTVIS [30]	2023	62.3	45.2	-	-
	VISAGE [41]	2024	64.6	46.7	-	-

**Table 11:** Parameters of the Jetson Nano Kit-B01.

Parameter	Specification
Size	100mm×80mm×29mm
CPU	4-Core ARM A57@1.43GHz
APU	Tegra X1
GPU	128-core Maxwell
Memory	32GB Micro SD Card
Display Interface	HDMI and DisplayPort
Graphics Memory	4GB 64-bit LPDDR4
Data Transmission Interfaces	4 × USB3.0, USB 2.0, Micro-B
ComputPerformance	473 GFlops

## Data Availability

The original data presented in the study are openly available in YouTube-VIS dataset at https://opendatalab.org.cn/OpenDataLab/YouTubeVIS2019, accessed on 23 March 2025. The UAV-VIS datset presented in this study are available on request from the corresponding author due to the privacy policy of the funder.
